# Surgical Management of a Pediatric Infratentorial Subdural Empyema as a Complication of Parapharyngeal Abscess

**DOI:** 10.7759/cureus.25270

**Published:** 2022-05-24

**Authors:** Boris Oleinikov, Gerald Musa, Matvey I Livshitz, Maria Kolcheva, Manuel de Jesus Encarnacion Ramirez, Renat Nurmukhametov, Ibrahim E Efe

**Affiliations:** 1 Neurosurgery, Peoples' Friendship University of Russia, Moscow, RUS; 2 Neurosurgery, Morozov Children's City Clinical Hospital of the Moscow Department of Health, Moscow, RUS; 3 Neurosurgery, Charité - Universitätsmedizin Berlin, Berlin, DEU

**Keywords:** pediatric brain abscess, posterior fossa empyema, parapharyngeal abscess, cerebellar abscess, subdural empyema, infratentorial empyema

## Abstract

Infratentorial empyema is a rare medical emergency typically presenting secondary to a middle ear infection. Nonspecific symptoms, limited access to radiological facilities, and imaging artifacts render this pathology prone to misdiagnosis and delayed intervention. An 11-year-old girl presented to the emergency department with a high fever, cervicalgia, and a two-week history of frontal headache. Computed tomography revealed parapharyngeal abscess and polysinusitis. Pus drained from the parapharyngeal abscess showed *Staphylococcus capitis* and *Streptococcus intermedius*. Treatment with intravenous meropenem and vancomycin led to initial improvement. On day five post drainage, she suddenly deteriorated with severe headache, vomiting, and posturing. Repeat CT showed posterior fossa empyema with hydrocephalus. The patient underwent an emergency suboccipital craniotomy for empyema evacuation. Pus cultures from the empyema showed identical results as those from the parapharyngeal abscess. Antibiotic therapy was continued for 12 weeks. The patient was discharged on day 21 after craniotomy with no neurological deficits. Early diagnosis and prompt neurosurgical evacuation combined with antibiotic therapy are of utmost importance to reduce morbidity and mortality. Physicians should consider the possibility of subdural empyema in children with parapharyngeal abscess and polysinusitis.

## Introduction

Infratentorial subdural empyema is a rare and life-threatening intracranial infection. Although subdural empyemas account for close to 20% of intracranial abscesses, only 1-10% are located in the posterior fossa [1-3]. They typically present as complications of otitis media, mastoiditis, sinusitis, or meningitis [4].

Their rarity, nonspecific symptoms, and the need for advanced radiological imaging make this pathology prone to delayed diagnosis [4-6]. Delayed treatment has been associated with high morbidity and mortality [7]. Hence, prompt diagnosis and immediate surgical evacuation combined with intravenous antibiotic therapy are of utmost importance [4,6-8]. The literature on infratentorial subdural empyema is scarce. To the best of our knowledge, no previous report exists on pediatric infratentorial subdural empyema as a complication of parapharyngeal abscess. The authors here present the successful treatment of an 11-year-old girl with posterior fossa empyema following a parapharyngeal abscess.

## Case presentation

**Figure 1:**
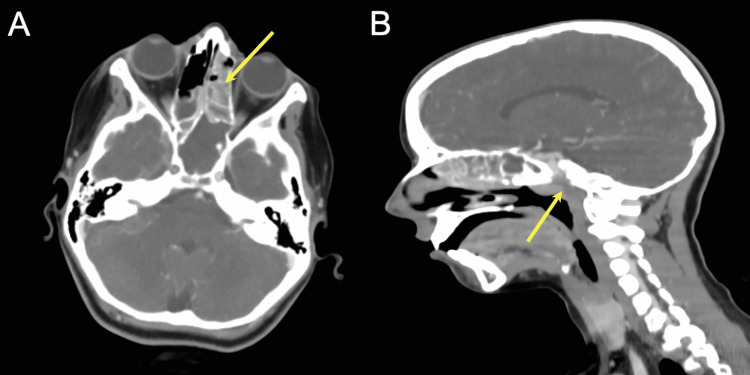
Initial CT scans showed polysinusitis in the axial image (A) and parapharyngeal abscess in the sagittal image (B).

An 11-year-old female presented to a local clinic with a three-day history of fever associated with frontal headache. The patient was given amoxicillin and antipyretic medication. Two weeks later, she returned with a 39.7°C (103.5°F) fever and cervicalgia. She was referred to the emergency department of our tertiary care center. CT scan revealed parapharyngeal abscess and acute sinusitis involving the maxillary, ethmoidal, and frontal air sinuses (Figure 1). A complete blood count showed severe leukocytosis (21.3 x 10*9/ml). The spinal tap showed slightly hazy cerebrospinal fluid (CSF) (Table 1). Based on the patient’s clinical presentation and elevated CSF white blood cell count, a diagnosis of bacterial meningitis was made.

**Table 1:** CSF analysis before pus drainage and on day four post drainage.

	Initial CSF analysis	Day 4 post drainage	Reference values
Appearance	Slightly turbid	Clear	Clear
White blood cell count	2230	0	<8/mm^3^
Protein	0.5	0.19	0.15-0.45 g/L

Antibiotic therapy was started with intravenous meropenem (750 mg) and vancomycin (0.75 g) in eight-hourly doses. The patient underwent endoscopic polysinusotomy and drainage of the parapharyngeal abscess. The pus collected from the sinuses was sterile. However, *Staphylococcus capitis* and *Streptococcus intermedius* were detected in the pus culture of the parapharyngeal abscess. Both pathogens showed sensitivity to ampicillin/sulbactam, vancomycin, linezolid, and meropenem. After pus drainage, the patient showed prompt improvement. Yet, mild cervicalgia and low-grade fever persisted. On day three post drainage, she had an elevated C-reactive protein (27 mg/l), elevated alpha-1 and alpha-2 globulins, and hypoalbuminemia (46 g/dl). CSF analysis was normal on day four post drainage.

**Figure 2:**
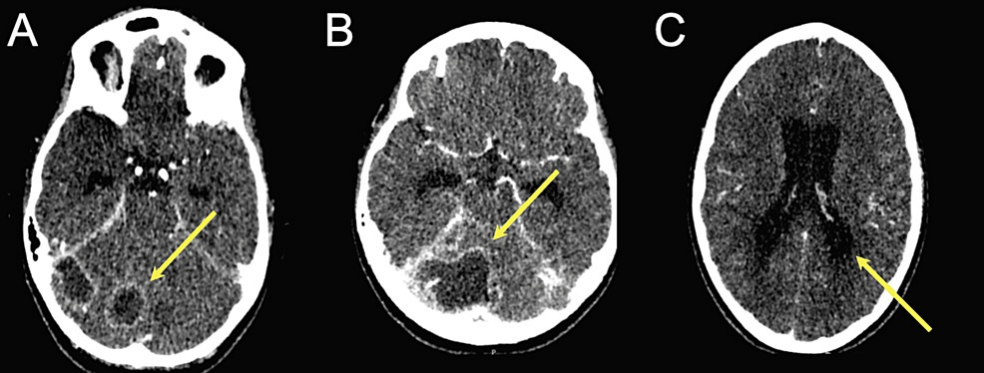
Axial CT scans revealed rim-enhanced lesions suggestive of abscess in the posterior fossa (A-B), causing hydrocephalus (C).

**Figure 3:**
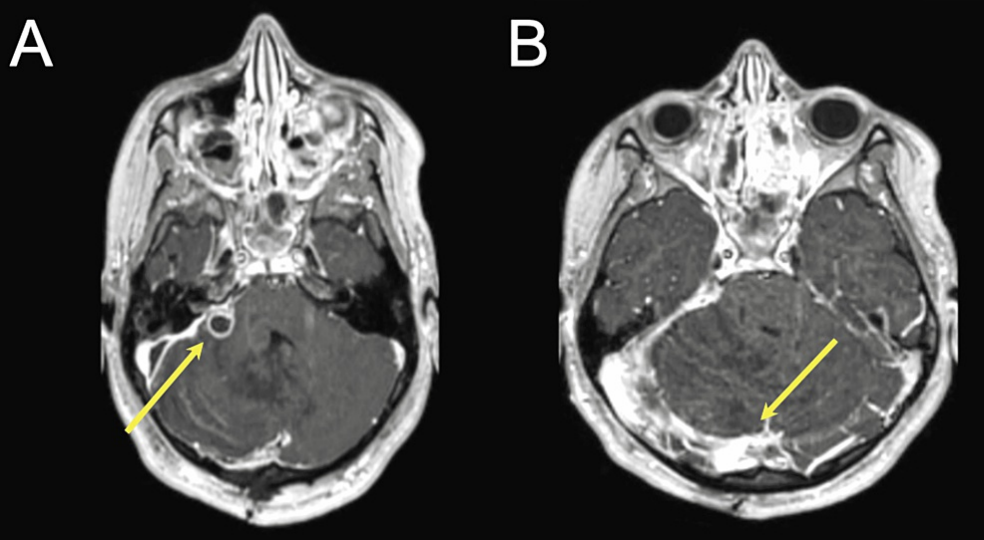
Contrast-enhanced T1-weighted MRI showed a small abscess in the right cerebellopontine angle (A) and thrombosis of the right transverse sinus (B).

On day five post drainage, the patient showed sudden deterioration with severe headache, posturing, and multiple episodes of projectile vomiting. Repeat head CT examination showed infratentorial subdural empyema of the posterior fossa associated with hydrocephalus (Figure 2). In contrast-enhanced T1-weighted MRI, a small abscess could be appreciated in the cerebellopontine (CP) angle. Further, thrombosis of the right transverse sinus was detected (Figure 3). Immediate surgical intervention was indicated. First, an external ventricular drain (EVD) was placed to lower intracranial pressure. A midline suboccipital craniotomy was then performed in the sitting position. The cerebellar surface and the tentorium were covered with fibrinous material. The infratentorial empyema was exposed. Its capsule extended to the pineal region encapsulating adjacent veins. Complete evacuation of the large empyema was successful with minimal blood loss. The small CP angle mass was not resected and assumed to regress with conservative antibiotic management. Intraoperative CSF cultures were sterile. Similar to the pus cultures of the parapharyngeal abscess, *Staphylococcus capitis* and *Streptococcus intermedius* were detected in pus drained from the empyema.

**Figure 4:**
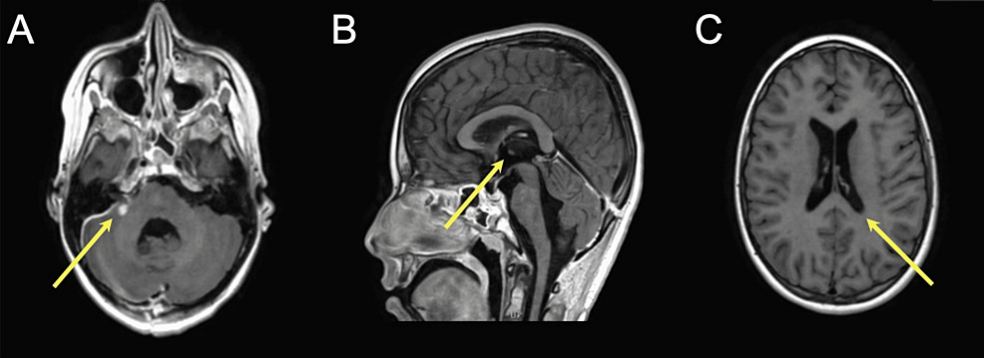
Repeat MRI on day 17 post-surgery showed regression of the cerebellopontine angle abscess (A) and hydrocephalus (B-C).

Meropenem was continued for four weeks (6 g daily) combined with linezolid (1200 mg daily). Subcutaneous sodium dalteparin (125 U/kg) was given subcutaneously for three months to treat the transverse sinus thrombosis. The EVD was removed on day seven post-surgery. Repeat MRI on day 17 post-surgery showed regression of both the CP angle abscess and the hydrocephalus (Figure 4). The patient was discharged on postoperative day 21 in good condition without any neurological deficits. Linezolid and dalteparin were continued for another two months.

## Discussion

Pediatric infratentorial subdural empyemas are an extremely rare medical emergency. The largest case series was published by Madhugiri et al. reporting 27 children with posterior fossa subdural empyema [9]. Seven patients underwent burr hole evacuation and 20 patients underwent craniotomy. Empyemas that were evacuated through burr holes only were highly prone to recurrence. Mean hospital stay was 25 days, hydrocephalus occurred in 20 patients, and one death was reported [9]. Further, five case reports described good recovery in children with infratentorial subdural empyema. In almost all cases, empyema occurred secondary to a middle ear infection [6,10-12].

Here, the patient presented with polysinusitis and a parapharyngeal abscess. The authors concluded that the parapharyngeal abscess was the most likely cause of subdural empyema as the pus cultures of both pathologies showed identical results. The proximity of the parapharyngeal space to the posterior fossa and the presence of extra- to intracranial venous drainage may explain this uncommon complication.

When first presenting to the local hospital, the patient was misdiagnosed and initial treatment was inappropriate. Nonspecific symptoms and inaccessibility of advanced imaging techniques pose a challenge to physicians in resource-limited centers in particular [3,4]. Further, the complex bony anatomy of the posterior fossa is prone to imaging artifacts rendering an accurate diagnosis of subdural empyema difficult [12]. By the time the diagnosis was made, the patient had developed cerebellar edema with tonsillar herniation and obstructive hydrocephalus leading to rapid neurological deterioration and emergency neurosurgery. Neurosurgical evacuation remains the first choice of treatment. Conservative strategies are associated with symptom relapse [4]. Cefotaxime and ceftriaxone are the most common first-line drugs for subdural empyema. Targeted antibiotic therapy necessitates pus collection and microbiological analysis [13,14].

## Conclusions

Children with infratentorial subdural empyemas can be treated successfully. Timely diagnosis and prompt surgical intervention combined with antibiotic therapy are crucial to reducing morbidity and mortality. Symptoms may be nonspecific and initial CT examination may be inconspicuous. Yet, physicians should consider subdural empyema as a differential diagnosis in children presenting with parapharyngeal abscess and polysinusitis.
